# Assessment of a New Formulation of Sildenafil on Common Practice: An Observational Study

**DOI:** 10.1155/2022/9122099

**Published:** 2022-06-02

**Authors:** Stéphane Droupy, Marie Hélène Colson

**Affiliations:** ^1^Urology and Andrology Department, Nimes University Hospital, Nimes, France; ^2^Immuno-Hematology Clinic (CISIH), Sainte-Marguerite Hospital, Marseille, France

## Abstract

Erectile dysfunction (ED) has a significant impact on the quality of life of patients. Xybilun® (IBSA Pharma SAS, France) is a new formulation of sildenafil in an orodispersible film (ODF). This study aims to assess the response rate (RR), satisfaction with, and safety of sildenafil-ODF in daily practice in France. Patients aged ≥18 years with ED were included in four groups: Group 1 mild, Group 2 moderate, Group 3 severe ED, according to the International Index for Erectile Function (IIEF)-6 subscore, never treated with phosphodiesterase inhibitors (PDE)5-I; Group 4, patients previously treated with another PDE5-I. Patients were evaluated at baseline (V1), one (V2), and three (V3) months. The RR and satisfaction were assessed using the IIEF-6 subscore questionnaire, a 5-point Likert scale, and a Global Assessment Question (GAQ). The primary endpoint for Groups 1 to 3 was the RR according to Rosen criteria at V3 compared to V1. For Group 4, the primary endpoint was the RR, defined as the satisfaction compared with previous treatment. Secondary endpoints were the RR at V2 compared to V1, the evolution of IIEF-6 and IIEF-15 scores, dose adjustment, satisfaction, convenience, and safety. One hundred and five patients were enrolled, 83 analysed. The RR at V3 was 100% (Group 1); 75% (Group 2); 65.2% (Group 3); and 84.2% (Group 4). The overall RR was 78.3%. Secondary parameters confirmed the satisfaction with sildenafil-ODF, with 81.6% of patients very satisfied at V3. No Serious Adverse Events (SAEs) were observed. In conclusion, sildenafil-ODF seems beneficial for patients irrespective of the severity of the ED. This study confirms in the context of daily clinical practice the satisfaction of patients with sildenafil-ODF. Data suggest that the availability of the intermediate dose of 75 mg could add greater flexibility to the therapy.

## 1. Introduction

Erectile dysfunction (ED) is defined by “The National Institutes of Health Consensus Development Conference on Impotence” as the persistent inability to attain and maintain an erection sufficient to permit satisfactory sexual performance [1, 2]. Recent European Association of Urology (EAU) guidelines established that ED is a frequent medical condition [1, 3]. The prevalence of ED is estimated at 52% between the ages of 40 and 70 [4], and at 19.3% between the ages of 30 and 80 with an increase from 2.3% to 53.4% [5]. The annual incidence ranges from 19.2 to 26 cases per thousand [6, 7]. ED has a significant impact on patients' quality of life (QoL) and their physical and psychosocial health. There is a growing body of scientific evidence demonstrating that ED is an early manifestation of peripheral vascular disease. ED should therefore be considered as a potential warning sign of cardiovascular disease [8], which is the leading cause of mortality. The management of ED includes the control of risk factors (tobacco use, obesity, sedentary lifestyle, chronic alcohol use, comorbidities, and depression) and appropriate pharmacological therapy [9]. The first-line treatment for ED is oral therapy with an inhibitor of cyclic guanosine monophosphate- (cGMP-) specific phosphodiesterase type 5 (PDE5-I). The PDE5-I results in intracellular accumulation of cGMP, which induces relaxation of smooth muscles and blood flow in the cavernous bodies of the penis. In this way, PDE5-I exerts a proerectile action helping to maintain the erection after sexual stimulation [8, 10]. Sildenafil is the first PDE5-I approved, and it represents an effective and safe oral drug treatment for ED [11, 12]. Xybilun® (IBSA Pharma SAS, France), a new orodispersible film formulation (sildenafil-ODF) available in four different dosage forms (25, 50, 75, and 100 mg) [13], represents a valid alternative to the tablets. The product is also available in other countries with the trade names Silandyl®, Rabestrom®, and Silvir®. Sildenafil-ODF is taken by placing the orodispersible film on the tongue. Sildenafil-ODF does not require the intake of a liquid; in comparison to coated-tablets, this mode of assumption is more subtle and unobtrusive [14]. The 75 mg dose allows a fine-tuned modulation of the therapy. Pharmacokinetic studies demonstrated that the sublingual and supralingual exposure to sildenafil-ODF is comparable to that of conventional sildenafil coated-tablets [15–17]. Observational studies are a valuable tool in the assessment of treatments for ED and a guidance for the daily clinical practice [18–20]. Patients and physician's evaluations of medicines are often different between randomised controlled trials (RCT) and real-life settings; this is mainly due to the strict inclusion/exclusion criteria, the frequent follow-up visits, and the close control of patients' compliance. Based on these considerations, the present multicentre observational study was aimed at collecting and comparing data in real-life setting use of sildenafil-ODF at different doses (50, 75, and 100 mg) in patients suffering from mild to severe ED during three months of treatment.

## 2. Materials and Methods

### 2.1. Study Objectives

This study was aimed at assessing the RR satisfaction of patients, physicians, partners, and safety of sildenafil-ODF in daily clinical practice. In real-world evidence studies, it is useful to have a feedback from the physician who is using the treatment on a daily basis. Therefore, evaluation of physician satisfaction with sildenafil-ODF was planned as a secondary parameter. The study collected descriptive information on the participating population (descriptive study) and then analysed the patients' response to the treatment with sildenafil-ODF (analytical study).

### 2.2. Study Design

This multicentre, prospective, longitudinal, observational study was carried out in France. Investigators were urologists and sexologists practicing in hospitals or as outpatient practices. Sixteen centres participated (inclusion of at least one patient). Patients were evaluated at baseline (V1), one (V2), and three (V3) months. The study was conducted in compliance with the Declaration of Helsinki and the French Code of Public Health (CSP). The study was approved by a national ethical committee (CPP Sud-ouest et Outre Mer-1). All patients gave their written informed consent. The study is recorded in (http://ClinicalTrial.gov/) repository (http://NCT04114240ClinicalTrial.gov/) [21].

## 3. Patients and Assessment Methods

Patients aged at least 18 years with mild, moderate, or severe ED with an International Index for Erectile Function (IIEF-6) subscore between 6 and 25 were enrolled in the study. Other inclusion criteria were as follows: patients who were informed about the study and gave free and informed statement of nonopposition; patients covered by social security. Exclusion criteria were as follows: known hypersensitivity to sildenafil or one of the excipients of sildenafil-ODF, severe cardiovascular disorders, and loss of vision in one eye due to anterior nonarterial ischemic optic neuropathy, regardless its causal relationship with previous exposure to PDE5-I. Patients treated with medicinal products containing nitric oxide donors, guanylate cyclase stimulators, alpha-blockers, and ritonavir in the three months before the selection were excluded. Patients were asked to have at least three monthly sexual activity attempts. Treatment with sildenafil-ODF was justified by the physician either as part of initial treatment for ED or in replacement of another similarly dosed therapy for ED. Patients were not enrolled if they had participated in another clinical trial, had a psychological or linguistic incapacity to understand the information sheet and under legal protection (guardianship, treatment), or deprived of their rights. Being the study aimed at the observation and collection of real life data, no placebo control group was available. Patients who accessed the clinical centres for a medical examination for ED were offered to participate in the study. The choice to treat the patients with sildenafil-ODF preceded and was independent of the patient's participation in the study. After the signature of the informed consent, the patients were included into four Groups (Group 1 to 3 naïve patients with mild to severe ED; Group 4 patients already treated for ED with another PDE5-I). The investigator prescribed sildenafil-ODF 50 mg to patients in Groups 1 to 3 according to the product labelling and a substitute dose to the previous PDE5-I in Group 4. It was estimated that physicians would recruit 8 patients each. The parties could mutually agree to increase or decrease the number, so that the overall recruitment would conform the required number of patients. Furthermore, in order for to balance the groups, the sponsor informed the physicians that they should not include patients in one or another group when the theoretical number of patients had been reached. The validated IIEF-15 tool, filled by the patients, was aimed at verifying the presence and severity of ED in a period of four weeks. In this study, six questions from IIEF-15 were used to assess erectile function (IIEF-6: items 1 to 5 and 15), evaluating the firmness of erection, the ability to penetrate, and maintain the hardness and confidence of the patient in maintaining an erection. The score for each question ranges from 1 (low sexual functioning) to 5 (the highest level of sexual functioning) [22–25]. A 5-point Likert scale (1, not at all satisfied; 2, slightly satisfied; 3, neutral; 4, satisfied; and 5, very satisfied) was used for the satisfaction assessment compared to prior treatment. Patients answered the question “Are you more satisfied with your current treatment than you were with your previous treatment?”

### 3.1. Parameters Explored by the Descriptive Study

The descriptive study collected data on demographic characteristics of the study population, on the results of the laboratory examinations recommended by the guidelines of the EAU [8], on the presence of risk factors and on the aetiology and clinical presentation of ED.

### 3.2. Endpoints of Analytical Study

The primary endpoint was the RR at V3 according to the efficacy criteria described by Rosen [24]. In detail, for Group 1 to 3, a patient was considered a responder if his IIEF-6 score between V1 and V3 increased by 2 points or more for Group 1, >5 points for Group 2, and >7 points for Group 3. The patients of Group 4 were considered responders if they rated 4 or 5 on a Likert scale to the question: “Are you more satisfied with your current treatment than you were with your previous treatment?” The secondary endpoints were RR at V2 and RR at IIEF-6 according to the severity and global change of IIEF-6. Satisfaction and convenience of sildenafil-ODF rated by patients, their partners, and physicians was evaluated using a 5-point Likert scale (1, not at all satisfied; 2, slightly satisfied; 3, neutral; 4, satisfied; and 5, very satisfied). Other secondary parameters assessed were the results of the IIEF-15 questionnaire and the dosage adjustment during the study. The safety was evaluated by active questioning about AEs at each visit.

### 3.3. Statistical Methods

Descriptive statistical analysis was performed on the characteristics of patients at baseline. All assessments were conducted in total and per subgroup (Group 1 to 4). A two-tailed test was used with a type I error (*α*) set at 5%. For the primary analysis, the response to treatment rate and the improvement in satisfaction compared to the previous treatment were described with a 95% confidence interval (95% CI). The explanatory factors of the binary response to treatment were determined by univariate analysis (Chi-square or Fisher tests): age, aetiology, body mass index, comorbidities, laboratory tests, etc, and using a step-by-step logistics model. The response was calculated globally according to the IIEF. An increase in 2, 5, and 7 points in Groups 1, 2, and 3, respectively, was considered as a response to treatment. The safety analysis was conducted using SAS version 9.4 (SAS Institute Inc., Cary, NC, USA).

## 4. Results

**Figure 1:**
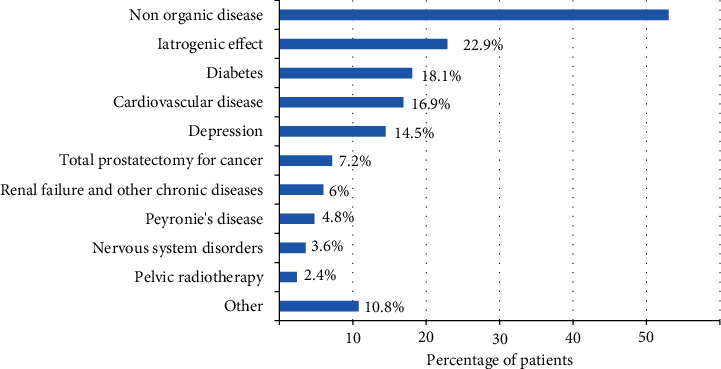
Aetiology of ED in the analysed population. The most frequent cause of ED is a nonorganic disease, followed by iatrogenic effects and diabetes.

A total of 105 patients were included between 10 July 2018 and 18 January 2019. Twenty-two of the 105 patients enrolled in the study (21%) were excluded from the analysed population due to major deviations from the study protocol but were included in the safety analysis. The analysed population included 83 patients: 13 patients (15.7%) in Group 1, 28 (33.7%) in Group 2, 23 (27.7%) in Group 3, and 19 (22.9%) in Group 4. The mean age of the study population was 57.4 years; 63.3% of the patients were obese or overweight. The most frequent laboratory abnormality was hypercholesterolemia (54.1%) followed by hypertriglyceridemia (32.4%) and hyperglycaemia (23.2%). Testosterone levels were considered normal in 77.5% of the patients (Table 1). The most common risk factor for ED was smoking (34.9%), followed by dyslipidaemia (22.9%) and alcoholism (14.5%). The most frequent aetiology of the ED was nonorganic (53%), followed by iatrogenic effects (22.9%) and diabetes (18.1%); 7.2% of the patients had previous prostatectomy or cystectomy for cancer, a group difficult to treat. The aetiology of ED in the study population is shown in Figure 1.

As for the clinical presentation of ED at inclusion in the global population, the patients' mean IIEF-6 score was 14.7 ± 6.3. Seventeen (20.5%) patients had mild ED; 38 (45.8%) had moderate and 28 (33.7%) had severe ED.

### 4.1. Primary Endpoint–Response Rate at Three Months

**Figure 2:**
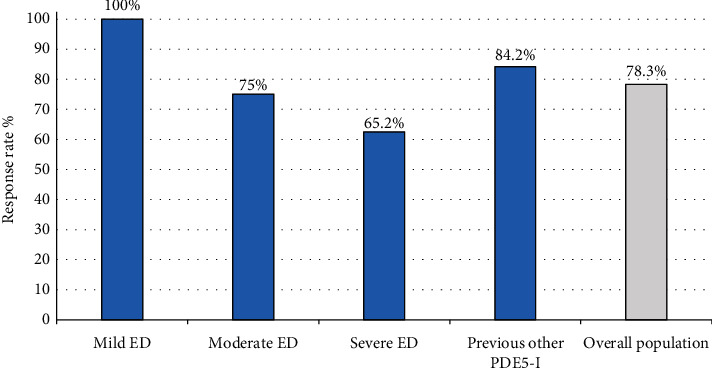
Response rate at V3 in each group and overall population. Group 1 (mild); Group 2 (moderate); Group 3 (severe) ED; Group 4 (previous treatment with other PDE5-Is).

The RR at V3 was 78.3% [95% CI 70.9; 85.8%] for the overall population. The RR at IIEF-6 was 100% in Group 1 (mild), 75% in Group 2 (moderate), and 65.2% in Group 3; 84.2% of patients of Group 4 were satisfied/very satisfied compared to the previous treatment (Figure 2).

Potentially predictive factors of response to treatment were tested in univariate analyses. Anejaculation was significantly negatively correlated (associated) with the response rate. The response rate was 50.0% in patients with anejaculation versus 83.1% in other patients (*p* = 0.0101). Noteworthy, a fair share (41.7%) of patients with anejaculation suffered from an ED following radical prostatectomy. Other factors, such as diabetes, depression, and severity of ED, are close or very close to the statistical significance (Table 2).

### 4.2. Secondary Analysis

**Figure 3:**
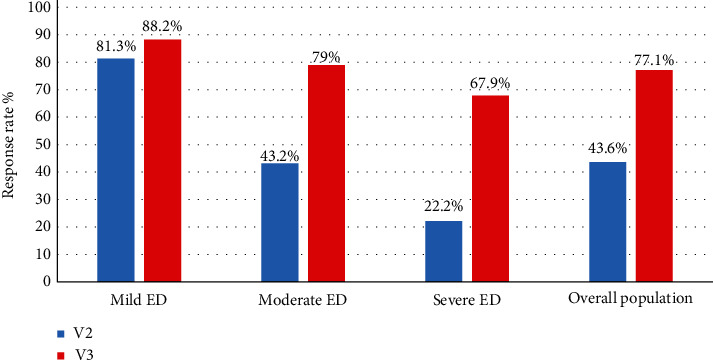
Response rate to the treatment in each group and in the overall population, based on IIEF-6 score, of Groups 1 (mild), 2 (moderate), and 3 (severe) at V2 and V3. V=Visit.

A total of 80 patients were evaluated at the 1-month visit (V2). The RR defined as the primary criteria at V2 was 48.8% for the entire analysed population [39.6%; 57.9%]. The RR at IIEF-6, based on the severity of ED, increased from V2 to V3. Consistently with the primary endpoint results, the RR was higher with mild and moderate ED; yet the improvement of RR was more marked in moderate and severe ED (Figure 3). The IIEF-6 score improved over time in all Groups. At V2, there was an average increase of 4.3 ± 4.0 points compared to V1, and the increase was more marked (+7.5 ± 4.6) at V3 compared to V1. The improvement observed is above the value of 4, defined as the Minimal Clinically Important Difference (MCID) in the IIEF-621. The repeated ANOVA measurements showed a significant (*p* < 0.0001) treatment effect over time regardless of the initial severity of ED. The ANOVA test did not show any statistical significant effect; the efficacy was the same independently from the treatment.

### 4.3. Improvement in the IIEF-15 Score during Follow-Up

The IIEF-15 score improved over time in all groups. Between inclusion and one month, the mean increase was 8.1 ± 8.0 points, while between enrollment and three months, the mean increase was more marked (+15.2 ± 10.1 points in the total population). All the components of the score (orgasmic function, sexual desire, intercourse satisfaction, and overall satisfaction) improved during follow-up. The between-group improvement in scores was tested statistically: all the components improved at a similar rate regardless of the patient group at inclusion, except for intercourse satisfaction, which increased more notably between inclusion and one month in Group 1 than in the other Groups (+2.9 points versus +1.6, +1.1 and +1.4 points in groups 2, 3, and 4, respectively).

### 4.4. Satisfaction and Convenience

**Figure 4:**
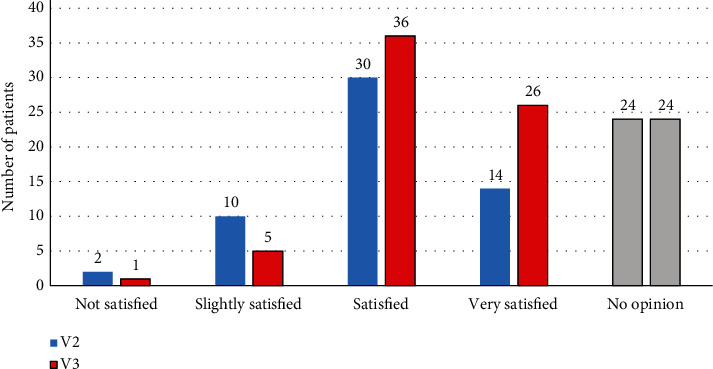
Number of patients satisfied with sildenafil-ODF at V2 and V3. V=Visit.

The vast majority of the study population was satisfied with the treatment: 61.5% and 81.6% of patients at V2 and V3, respectively, reported a better quality of the erection. The percentage of patients reporting an improvement in the ability to engage in sexual activity was 51.4% at V2 and 82.2% at V3. This positive result was independent of the response to the treatment. Regarding the overall satisfaction with sildenafil-ODF, 55.0% and 81.6% of patients were satisfied or very satisfied at V2 and V3, respectively. The proportion of patients who found the treatment convenient/very convenient at V2 and V3 was, respectively, 73.4% and 81.3%. The following graph shows the level of satisfaction of the patient population, expressed in number of patients (Figure 4). The above results were observed regardless of response to treatment. In comparison, at the time of inclusion, among the 19 patients previously treated with other PDE5-Is (Group 4), five patients (26.3%) were satisfied.

Physician satisfaction levels improved during the follow-up: 15.0% were slightly or not satisfied at one month compared to 7.9% at three months, while 55.0% and 81.6% were satisfied/very satisfied at one month and three months, respectively. Out of 36 responders' partners, 25 (69.4%) were satisfied/very satisfied, 5 (13.9%) were slightly or not satisfied, and 6 (16.7%) were neither satisfied nor dissatisfied.

### 4.5. Treatment Adjustments

At V1, 81.9% of the patients was given a 50 mg dose. In the majority of patients, the dose was increased to 75 mg and 100 mg at V2 and V3 (Table 3). At V2, 27.8% of patients was treated at 75 mg and this percentage remained similar (25.8%) at V3; it can be concluded that the availability of the 75 mg prevented in these patients the increase to 100 mg (Table 3). In total, of the 83 patients analysed, 64 patients (77.1%) continued treatment with sildenafil-ODF, 40 patients (48.1%) changed doses, and five patients (6.0%) took it together with another treatment. After switching to sildenafil-ODF (Group 4), 16 of the 19 patients in Group 4 (84.2%) continued taking sildenafil-ODF, ten patients (52.6%) changed doses, and two patients (10.5%) received sildenafil-ODF together with another treatment.

### 4.6. Safety

Safety was studied in the total population composed of 105 patients. Thirty-two patients (30.5%) experienced a total of 72 AEs: 62 AEs occurred in 27 patients (33.8%) treated for the first time for ED and 10 AEs occurred in 5 previously treated patients (21.7%). The most-reported AE was headache (32 AEs in 18 patients; 17.1%) followed by nasal congestion (9 AEs in 6 patients; 5.7%), flushing (6 AEs in 5 patients; 4.8%), and hot flush (4 AEs in 3 patients; 2.9%). The reported data are consistent with the known safety profile of the product, as the most frequent AEs were those listed in the summary of product characteristics.

## 5. Discussion

The main objective of this study was to perform a descriptive and analytical assessment of the RR, safety, and patients and physicians' satisfaction with a new formulation of sildenafil-ODF (Xybilun®, IBSA Pharma SAS, France) in a real-life setting. This study shares the limits of other observational studies in terms of sample size achieved, difficulty to assess the compliance of patients, details of information, and some missing data. Moreover, observational studies cannot use multiple and complex assessment tools for patients as RCTs do. Indeed, the observational studies' approach is to interfere as little as possible in the daily clinical practice of recruited patients, e.g., by avoiding or limiting the use of control groups treated with placebo or the use of multiple assessment scales. However, the above-mentioned limits are outweighed by information on patients' behaviour in real life. The baseline characteristics of the study population were similar to those of patients enrolled in previous studies on PDE5-Is [19, 20], and the comorbidities identified in the population were similar to what described in the scientific literature. The above evidence supports that the study population was representative of the general population affected by ED [17, 23]; the results of this study can, therefore, be considered as valid for the general population of patients using PDE5-Is for ED. The descriptive data confirmed the role of nonorganic factors as the most frequent cause of ED. At this regard, future approaches could also consider performing a subset analysis of men who presented with nonorganic ED. Iatrogenic and organic origins of ED are also frequent and should always be investigated by the physician during the first assessment of a patient complaining of ED [26–28]. Attention should be paid to the risk factors, like smoking and dyslipidaemia, confirming the opportunity to consider lifestyle modification as reported in literature [29]. The RR to the treatment at V3 (primary endpoint) was satisfactory: eight out of 10 patients responded to the treatment. The RR was higher in the milder cases at treatment initiation, although symptoms improved regardless of the initial severity of ED. The RR of sildenafil-ODF was not affected by prior treatment, since previously treated patients in Group 4 had a RR close to that of the whole cohort. The analysis of factors predictive of inadequate response provided interesting results: anejaculation was statistically associated with inadequate response to the treatment; this is probably due to the fact that 41.7% of these patients had a positive history for radical prostatectomy, which makes them very difficult to treat. Diabetes, depression, iatrogenic ED, and severity of the clinical manifestations of ED were associated to a lower efficacy of the therapy without reaching statistical significance. Age, however, did not emerge as a risk factor for treatment failure. The results of secondary endpoints indicate that, even if the first effects occur at one month, the peak of efficacy is reached probably around the third month of treatment. The increase of effectiveness at three months could be due, at least partially, to the dose adjustment, especially in those with the most severe symptoms. Effects were similar in patients previously treated for ED and in those who just started treatment. Virtually, one in three patients of the entire cohort was relieved entirely and no longer presented ED symptoms at three months. Fewer than one in ten patients still showed severe symptoms against one in three at inclusion. Consistently, with the primary endpoint, the improvement of the symptoms defined by the IIEF-6 score RR is higher in mild/moderate groups. Although this is a real life setting performed study, and hence not strictly comparable with RCT's, this result was in agreement with the results of previous RCTs [23]. It is noteworthy that the severity did not impact the response to IIEF-6. The results of the IIEF-15 questionnaire complemented the results of the IIEF-6 subscore showing improvement to all the dimensions explored by the tool (orgasmic function, sexual desire, intercourse satisfaction, and overall satisfaction). Last but not least, in a real-life study, the patients' satisfaction is of paramount importance. Patients expressed a high level of satisfaction (81.6%), and also, half of the nonresponders reported to be satisfied, which suggests that the treatment at least partially met their expectations. Only a few patients switched to another treatment during follow- up, except in the group with severe ED. Most patients continued treatment with sildenafil-ODF with dose adjustments. Furthermore, the study confirmed the safety profile of sildenafil-ODF with the most commonly reported AEs being those listed in the summary of the product's characteristics [13]. It is also to be stressed that while PDE5-I are among the most counterfeited medication, the specific ODF formulation appears virtually exempt from the risk of counterfeiting [30]. This study, like other real-life studies, has several limitations. For example, the follow-up is relatively short; there are, beside testosterone, additional hormones (including prolactin, LH, and TSH) not determined here that could have relevance in the ED development, as outlined in the review by Sansone et al. [31]. Beside these limitations, we think that this study reinforces the activity of sildenafil-ODF showing its efficacy in this particular setting.

## 6. Conclusions

In conclusion, this study confirms, in the context of daily clinical practice, the satisfaction of patients with sildenafil-ODF. Data also suggest that the availability of the intermediate dose of 75 mg could add greater flexibility to the therapy.

## Figures and Tables

**Table 1:** Characteristics and laboratory parameters of the study population. The laboratory values of patients were classified as “normal” or “abnormal” according to the reference values of each centre.

Age (mean ± SD)		57.4 ± 18.0
SBP (mean ± SD)		104 ± 50
DBP (mean ± SD)		67.1 ± 30.3
BMI	N	79
Missing	N	4
Obese	N (%)	27 (22.8%)
Overweight	N (%)	32; (40.5%)
Fasting blood sugar	N	69
Missing	N	14
Hyperglycaemia	N (%)	16 (23.2%)
Normal	N (%)	53 (76.8%)
HbA1c	N	60
Missing	N	23
<7	N (%)	17 (73.9%)
>7.5	N (%)	3 (13%)
[7; 7.5]	N (%)	3 (13%)
Total cholesterol	N	74
Missing	N	9
Abnormal	N (%)	40 (54.1%)
Normal	N (%)	34 (45.9%)
LDL cholesterol	N	71
Missing	N	12
Abnormal	N (%)	20 (28.2%)
Normal	N (%)	51 (71.8%)
HDL cholesterol	N	73
Missing	N	10
Abnormal	N (%)	9 (12.3%)
Normal	N (%)	64 (87.7%)
Triglycerides	N	74
Missing	N	9
Abnormal	N (%)	24 (32.4%)
Normal	N (%)	50 (67.6%)
Testosterone	N	71
Missing	N	12
Abnormal	N (%)	16 (22.5%)
Normal	N (%)	55 (77.5%)

**Table 2:** Characteristics and laboratory parameters of the study population. The laboratory values of patients were classified as “normal” or “abnormal” according to the reference values of each centre.

Variables	Grade	Nonresponders (*N* = 18)	Responders (*N* = 65)	*p* value (2-sided)
Anejaculation	No	12 (16.9%)	59 (83.1%)	0.0101^a^
	Yes	6 (50%)	6 (50%)	
Diabetes	No	12 (17.6%)	59 (83.1%)	0.0572^b^
	Yes	6 (40%)	6 (50%)	
ED	Mild ED	1 (5.9%)	16 (94.1%)	0.0593^a^
	Moderate ED	7 (18.4%)	31 (81.6%)	
	Severe ED	10 (35.7%)	18 (64.3%)	
Iatrogenic effect (antihypertensives, psychotropics)	No	11 (17.2%)	53 (82.8%)	0.0679^b^
	Yes	7 (36.8%)	12 (63.2%)	
Depression	No	13 (18.3%)	58 (81.7%)	0.0694^b^
	Yes	5 (41.7%)	7 (58.3%)	

^a^Fisher's test; ^b^Chi-square test.

**Table 3:** Percentage of patients assuming 100, 75, or 50 mg of sildenafil-ODF at the different visits.

Dosage (mg)	V1	V2	V3
	% of patients		
100	6.7	36.7	45.2
75	11.0	27.8	25.8
50	81.9	35.4	29.0

## Data Availability

The clinical data used to support the findings of this study are available from the corresponding author upon request.
